# Thymic CT attenuation score in SAPHO syndrome: a comparative study with rheumatoid arthritis, ankylosing spondylitis, and healthy controls

**DOI:** 10.3389/fimmu.2026.1846112

**Published:** 2026-09-28

**Authors:** Fengyu Liu, Liujie Zheng, Tao Wang, Ling Wang, Lin Liu, Erdong Zhang, Yongle Wei, Sujuan Xu, Ce Wang, Feng Luan, Zhiyong Hou

**Affiliations:** 1 Hebei Medical University Clinical Medicine Postdoctoral Research Station (Hebei Medical University Third Hospital), Shijiazhuang, Hebei, China; 2 Department of Orthopedic Surgery, Hebei Medical University Third Hospital, Shijiazhuang, Hebei, China; 3 Orthopaedic Research Institute of Hebei Province, Shijiazhuang, Hebei, China; 4 Department of Lower Limb Trauma, Beijing Jishuitan Hospital, Guizhou Hospital, Guiyang, Guizhou, China; 5 Department of Nephrology, Hebei Medical University Third Hospital, Shijiazhuang, Hebei, China; 6 Department of Otorhinolaryngology, Hebei Medical University Third Hospital, Shijiazhuang, Hebei, China; 7 Key Laboratory of Biomechanics of Hebei Province, Shijiazhuang, Hebei, China; 8 NHC Key Laboratory of Intelligent Orthopaedic Equipment, Shijiazhuang, Hebei, China; 9 Engineering Research Center of Orthopedic Minimally Invasive Intelligent Equipment, China Ministry of Education, Shijiazhuang, Hebei, China; 10 Key Laboratory of Precise Assessment, Diagnosis, and Treatment of Soft Tissue Injury of Hebei Province, Shijiazhuang, Hebei, China

**Keywords:** ankylosing spondylitis, computed tomography, rheumatoid arthritis, SAPHO syndrome, thymic attenuation, Thymus

## Abstract

**Objective:**

SAPHO syndrome is a rare autoinflammatory/autoimmune overlap disorder whose thymic imaging characteristics remain unexplored. This study evaluated thymic CT attenuation scores in patients with SAPHO syndrome compared with rheumatoid arthritis (RA), ankylosing spondylitis (AS), and healthy controls (HC).

**Methods:**

This retrospective study included 180 patients with SAPHO syndrome, 60 with RA, 60 with AS, and 180 HC, all aged ≥30 years. Thymic attenuation was assessed using a 4-point scale (0–3) and CT attenuation values in Hounsfield units (HU). Higher attenuation was defined as score ≥2. Subgroup analyses were performed by sex and age (<45 vs ≥45 years).

**Results:**

The prevalence of higher thymic attenuation differed significantly among the four groups (P<0.001), with proportions of 31.1% in SAPHO, 25.0% in RA, 8.3% in AS, and 10.6% in HC. *Post hoc* analyses of the ordinal attenuation score and CT attenuation values showed higher values in SAPHO and RA than in AS and HC, whereas SAPHO and RA did not differ significantly from each other. This pattern appeared most pronounced among women aged <45 years, in whom higher attenuation was observed in 54.3% of SAPHO and 47.8% of RA patients, compared with 19.2% of HC and 3.8% of AS patients. Within the SAPHO group, higher attenuation was associated with female sex (85.7% vs 68.5%, P = 0.015) and younger age (median 37.5 vs 44 years, P<0.001).

**Conclusion:**

Thymic CT attenuation was higher in SAPHO and RA, particularly among women aged <45 years, whereas AS showed findings similar to HC. Increased thymic attenuation may represent a potential imaging phenotype associated with SAPHO syndrome and RA. Further longitudinal and pathological studies are needed to clarify its biological significance.

## Introduction

The thymus is a central lymphoid organ essential for T-cell development and the establishment of central tolerance, and its structural and functional abnormalities have been closely linked to various autoimmune diseases (1, 2). In myasthenia gravis (MG), thymic follicular hyperplasia is strongly correlated with anti-acetylcholine receptor antibody titers, with 80% of affected patients being female and the peak onset occurring between 20 and 40 years of age (3). In rheumatoid arthritis (RA), a high thymic attenuation score (≥2) has been reported in 12.9% of patients and is strongly associated with positivity for anti-citrullinated protein antibodies (ACPA) (odds ratio = 15.2) (4). In systemic sclerosis (SSc), thymic hyperplasia (>13 mm) and incomplete involution (>7 mm) have been observed in 9.5% and 15% of patients, respectively, and have been associated with younger age and the diffuse cutaneous subset (5, 6). In primary Sjögren’s syndrome (pSS) and polymyositis/dermatomyositis (PM/DM), the proportions of patients with high thymic attenuation are 15.3% and 19.1%, respectively (7).

In the general population, significant sex- and age-related differences in thymic CT appearance have been documented. Ackman et al. (8) reported that women aged 20–30 years have significantly higher thymic attenuation than men of the same age and more often exhibit a quadrilateral morphology. Naik et al. (9) reported that the mean age for complete fatty replacement of the thymus is 45.4 years. These normal variations underscore the importance of accounting for sex and age when interpreting thymic imaging findings in disease states.

SAPHO syndrome (synovitis, acne, pustulosis, hyperostosis, and osteitis) is a rare autoinflammatory/autoimmune overlap disorder characterized by aseptic inflammatory osteitis, synovitis, and cutaneous lesions (10, 14). The Th17/IL-17 pathway has also been implicated in its pathogenesis (13). To date, no studies have evaluated thymic imaging findings in patients with SAPHO syndrome. Given the central role of the thymus in immune tolerance, the reported thymic imaging abnormalities in several autoimmune diseases, and the known influence of age and sex on normal thymic appearance, thymic imaging characteristics in SAPHO syndrome warrant investigation.

To address this gap, the present study was designed to systematically assess thymic CT attenuation scores in patients with SAPHO syndrome for the first time and to compare them with those in patients with RA, AS, and healthy controls. AS was included as a disease comparator because it is another chronic inflammatory rheumatic disease with prominent IL-23/IL-17-related mechanisms but a substantially different immunopathogenic profile from RA (15–18). We also explored whether group differences in thymic CT findings varied according to age and sex.

## Methods

### Study design

This retrospective study was approved by the hospital ethics committee, and informed consent was waived.

### Study population

This study employed a stratified frequency-matched case–control design. A total of 180 patients with SAPHO syndrome who were treated at our hospital between January 2023 and January 2026 were initially enrolled. The diagnosis was established according to the criteria proposed by Kahn et al. (12, 20), and all patients demonstrated characteristic findings on bone scintigraphy, including increased radiotracer uptake in the sternal region, which is a hallmark feature of SAPHO syndrome (21). Using the age and sex distribution of the SAPHO group as a reference, 180 healthy controls (with no history of autoimmune diseases or thymic disorders) were selected using stratified frequency matching. In addition, 60 patients with rheumatoid arthritis (RA) who met the 2010 American College of Rheumatology/European League Against Rheumatism classification criteria for rheumatoid arthritis were enrolled (22). Similarly, 60 patients with ankylosing spondylitis (AS) who fulfilled the modified New York criteria were included (23). The RA and AS groups were selected to achieve sex and age distributions comparable to those of the SAPHO group (**Table 1**). All participants were aged ≥30 years, based on reports that thymus gland involution is not completed until age 30 years (4). Exclusion criteria included age <30 years, thymoma, thymic cyst, and malignancy.

**Table 1:** Baseline characteristics and comparison of thymic CT findings among the four groups.

Variable	SAPHO (n=180)	RA (n=60)	AS (n=60)	HC (n=180)	*P*
Female, n(%)	133(73.9%)	44(73.3%)	44(73.3%)	133(73.9%)	0.999
Age, years, median (IQR)	42(36, 50)	42(36, 50)	42(36.3, 50.8)	43(37, 49.8)	0.883
Disease duration, months, median (IQR)	24.0 (12.0, 60.0)	36.0 (24.0, 96.0)	120.0 (36.0, 228.0)	NA	–
Glucocorticoid use, n (%)	11 (6.1%)	7 (11.7%)	3 (5.0%)	NA	–
csDMARD use, n (%)	9 (5.0%)	30 (50.0%)	31 (51.7%)	NA	–
Biologic/targeted synthetic DMARD use, n (%)	41 (22.8%)	19 (31.7%)	32 (53.3%)	NA	–
Mean CT attenuation, HU, median (IQR)	-44.6(-61.4, -17.6)	-37.9(-55.9, -18.3)	-66.4(-77.5, -39.4)	-64.5(-77.3, -47.7)	<0.001
Thymic attenuation score, n(%)					<0.001
Score 0	43(23.9%)	11(18.3%)	33(55.0%)	93(51.7%)	
Score 1	81(45.0%)	34(56.7%)	22(36.7%)	68(37.8%)	
Score 2	41(22.8%)	9(15.0%)	4(6.7%)	16(8.9%)	
Score 3	15(8.3%)	6(10.0%)	1(1.7%)	3(1.7%)	
Higher attenuation (Score 2-3), n(%)	56(31.1%)	15(25%)	5(8.3%)	19(10.6%)	<0.001

IQR, interquartile range; SAPHO, synovitis, acne, pustulosis, hyperostosis, osteitis; RA, rheumatoid arthritis; AS, ankylosing spondylitis; HC, healthy controls; HU, Hounsfield units; csDMARDs, conventional synthetic disease-modifying antirheumatic drugs; NA, not applicable.

Patients with SAPHO syndrome, RA, and AS were hospitalized patients, and chest CT was performed as part of routine inpatient clinical evaluation. Healthy controls underwent chest CT as part of routine health examinations at the hospital health examination center. None of the CT examinations was performed specifically for evaluation of the thymus.

### Imaging assessment

All participants underwent non-contrast chest CT using a Siemens SOMATOM Definition scanner (Siemens, Germany). Images were acquired at a tube voltage of 120 kV with automatic tube-current modulation and transferred to the picture archiving and communication system (PACS) for analysis. Thymic visual scoring and quantitative CT attenuation measurements were performed on axial images reconstructed at a slice thickness of 1.0 mm using the B30f kernel. Thymic attenuation was independently assessed by two observers who were blinded to clinical information using a previously described four-point scale (4, 8) (**Figure 1**): score 0, complete fatty degeneration; score 1, predominantly fat density; score 2, half solid tissue and half fat attenuation, or solid tissue >7 mm; and score 3, predominantly solid tissue. Higher attenuation was defined as scores 2–3 and lower attenuation as scores 0–1. In cases of disagreement, the two observers reviewed the images together and reached a consensus, and the consensus score was used for subsequent analysis.

**Figure 1:**
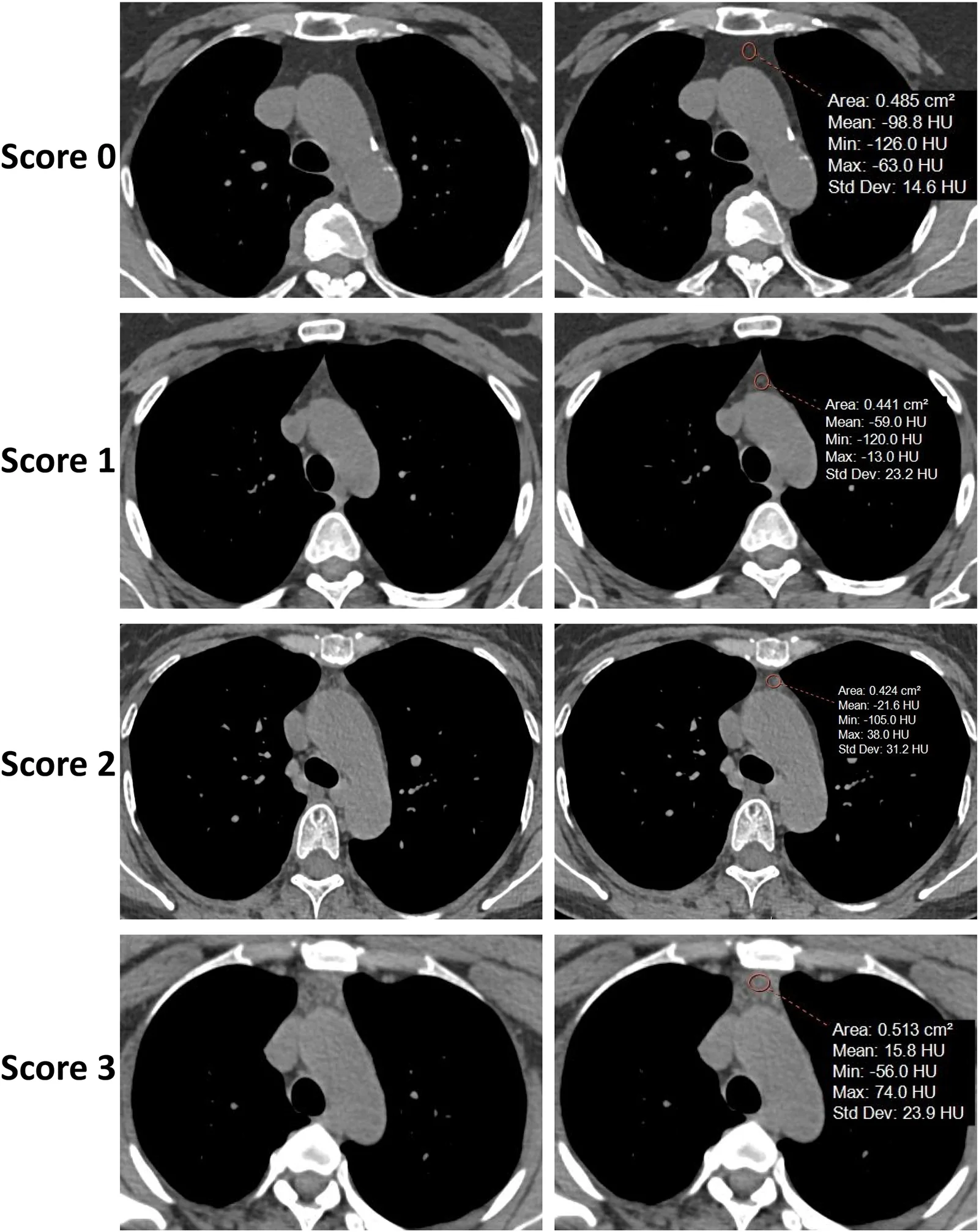
Representative axial chest CT images illustrating the four-point thymic attenuation scoring system and ROI placement. The left panels show the original CT images, and the right panels show the corresponding images with region-of-interest (ROI) placement for CT attenuation measurement. According to the previously described four-point scale (4, 8), score 0 indicates complete fatty degeneration; score 1, predominantly fat density; score 2, half solid tissue and half fat attenuation, or solid tissue >7 mm; and score 3, predominantly solid tissue. The right panels show the corresponding ROI placement and CT attenuation measurements (Hounsfield units, HU).

CT attenuation values (Hounsfield units, HU) were independently measured by two observers on axial images at three representative levels of the thymic region. For participants with identifiable thymic tissue (thymic scores 1–3), a circular region of interest (ROI) of approximately 0.5 cm² was placed within the thymic boundaries, with the ROI size adjusted when necessary according to thymic size. For participants with complete fatty replacement (thymic score 0), ROIs were placed within the expected anatomical thymic bed based on its typical location in the anterior mediastinum. Blood vessels, adjacent non-thymic structures, artifacts, and areas affected by partial-volume effects were carefully avoided. For each observer, the mean HU value of the three ROIs was calculated. The average of the mean values obtained by the two observers was used as the final thymic CT attenuation value for statistical analysis.

### Clinical data collection

For patients with SAPHO syndrome, RA, and AS, disease duration, defined as the interval from the onset of the first disease-related symptom to the chest CT examination, and immunomodulatory/immunosuppressive treatments being received at the time of chest CT were recorded. Treatment categories included systemic glucocorticoids, conventional synthetic disease-modifying antirheumatic drugs (csDMARDs), and biologic or targeted synthetic DMARDs. For the SAPHO group, additional clinical data included symptom type (cutaneous lesions, bone pain, or both), smoking status, alcohol consumption, C-reactive protein (CRP) level, and erythrocyte sedimentation rate (ESR).

### Statistical analysis

Statistical analyses were performed using SPSS version 21.0 (IBM Corp., Armonk, NY, USA). Continuous variables are presented as median (interquartile range [IQR]) and were compared among the four groups using the Kruskal–Wallis test. When the overall test was significant, pairwise comparisons were performed using the Mann–Whitney U test with Bonferroni correction for six comparisons. Disease duration and treatment variables were summarized descriptively. The thymic CT attenuation score (0–3) was treated as an ordinal variable and analyzed using the same nonparametric approach. Categorical variables are presented as number (percentage) and were analyzed using the Pearson chi-square test; Fisher’s exact test was used when expected cell counts were small. Within the SAPHO group, continuous variables were compared between the higher- and lower-attenuation subgroups using the Mann–Whitney U test, and categorical variables were compared using the Pearson chi-square test or Fisher’s exact test, as appropriate. All tests were two-sided, and P<0.05 was considered statistically significant; for pairwise comparisons, statistical significance was determined using Bonferroni-adjusted P values. Interobserver agreement for the 4-point thymic attenuation scale was assessed using quadratic-weighted κ based on the initial independent scores obtained before consensus. Agreement for CT attenuation values was assessed using ICC(2,1) (two-way random-effects, absolute agreement) based on the independent measurements obtained from the two observers before averaging.

## Results

### Baseline characteristics

A total of 480 participants were enrolled, comprising 180 patients with SAPHO syndrome, 60 with RA, 60 with AS, and 180 healthy controls (HC). The four groups had comparable sex and age distributions, consistent with the frequency-matching strategy. The proportion of female participants was 73.9% in the SAPHO group, 73.3% in the RA group, 73.3% in the AS group, and 73.9% in the HC group, with no significant difference among groups (P = 0.999). Median ages were 42 years in the SAPHO group, 42 years in the RA group, 42 years in the AS group, and 43 years in the HC group (P = 0.883) (**Table 1**). Interobserver agreement was excellent for both the 4-point thymic attenuation scale (quadratic-weighted κ = 0.954, 95% CI: 0.938–0.970) and CT attenuation values (ICC(2,1) = 0.925, 95% CI: 0.911–0.937).

### Comparison of thymic CT attenuation scores and CT values among the four groups

Thymic CT attenuation scores and CT attenuation values differed significantly among the four groups (both P<0.001). The prevalence of higher attenuation (score ≥2) also differed significantly across groups (P<0.001), with proportions of 31.1% (56/180) in SAPHO, 25.0% (15/60) in RA, 8.3% (5/60) in AS, and 10.6% (19/180) in HC (**Table 1**).

*Post hoc* comparisons showed that the SAPHO and RA groups each had higher attenuation scores and higher CT attenuation values than the AS and HC groups (all Bonferroni-adjusted P<0.01), whereas no significant differences were observed between SAPHO and RA or between AS and HC. Median CT attenuation values were −44.6 HU in SAPHO, −37.9 HU in RA, −66.4 HU in AS, and −64.5 HU in HC. The distributions of attenuation scores and CT attenuation values across the four groups are illustrated in **Figure 2**.

**Figure 2:**
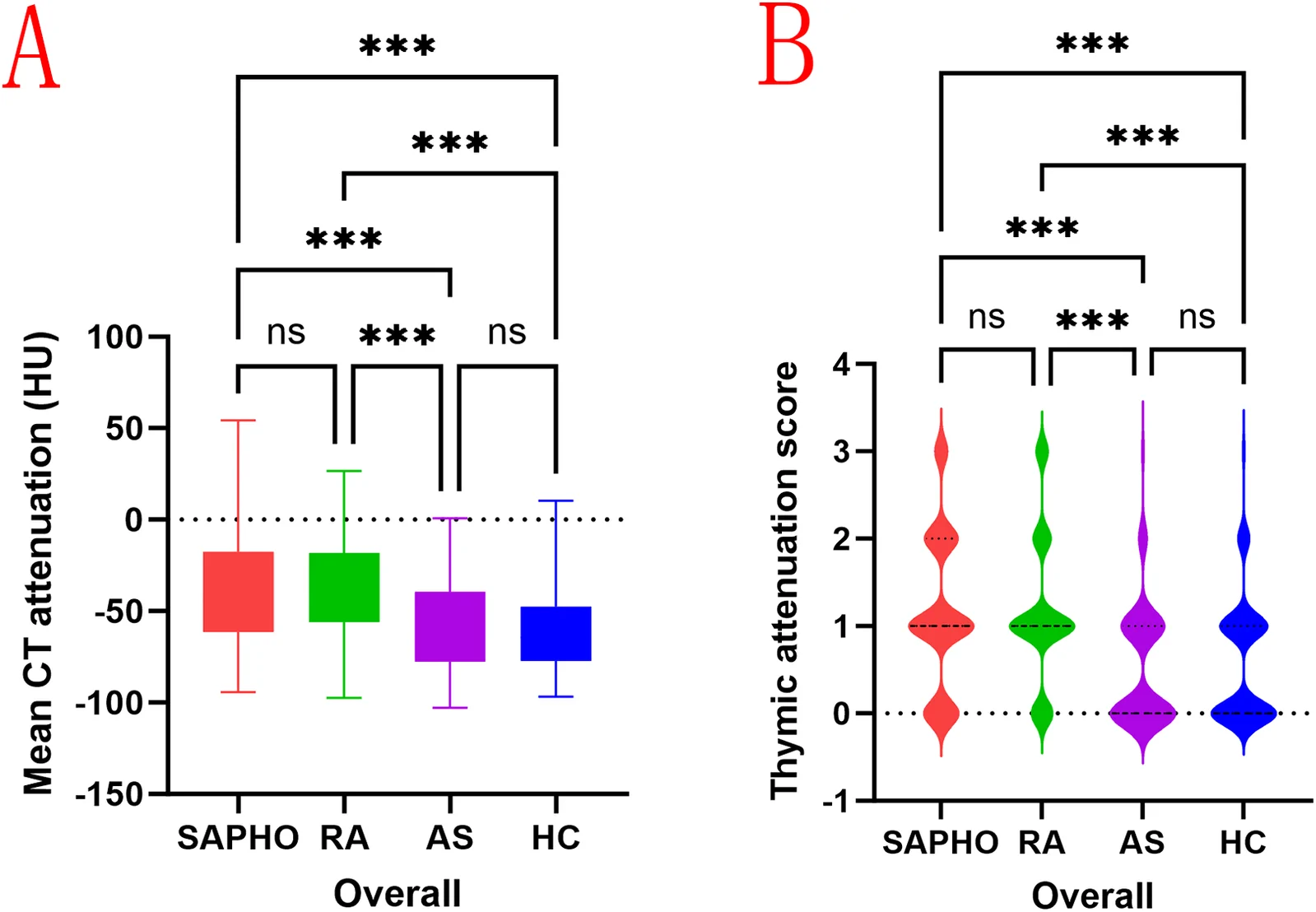
Comparison of thymic CT attenuation values and attenuation scores among the four groups. **(A)** Box-and-whisker plots showing the distribution of CT attenuation values (HU). **(B)** Violin plots showing the distribution of thymic attenuation scores (0–3). Pairwise comparisons were performed using the Mann–Whitney U test with Bonferroni correction. ***Bonferroni-adjusted P<0.001, **P<0.01, *P<0.05; ns, not significant; SAPHO, synovitis, acne, pustulosis, hyperostosis, osteitis; RA, rheumatoid arthritis; AS, ankylosing spondylitis; HC, healthy controls; HU, Hounsfield units.

### Sex- and age-stratified analysis of thymic CT findings

To further investigate the influence of sex and age on thymic CT findings, participants were stratified into four subgroups based on sex (female/male) and age (<45 years vs. ≥45 years), using 45 years as the cut-off based on the mean age of complete fatty replacement in the general population (9). The results of the sex- and age-stratified analysis are summarized in **Table 2** and **Figures 3**, **4**.

**Table 2:** Sex- and age-stratified analysis of thymic CT findings.

Subgroup	Variable	SAPHO	RA	AS	HC	*P*
Female <45 years	Mean CT attenuation, HU, median (IQR)	-27.7(-47.4, -5.1)	-20.5(-44.7, 2.0)	-66.4(-76.6, -37.1)	-52.7(-68.6, -36.5)	<0.001
	Thymic attenuation score, n(%)	n = 81	n = 23	n = 26	n = 73	<0.001
	Score 0	11(13.6%)	2(8.7%)	15(57.7%)	23(31.5%)	
	Score 1	26(32.1%)	10(43.5%)	10(38.5%)	36(49.3%)	
	Score 2	30(37.0%)	5(21.7%)	0(0%)	11(15.1%)	
	Score 3	14(17.3%)	6(26.1%)	1(3.8%)	3(4.1%)	
Female ≥45 years	Mean CT attenuation, HU, median (IQR)	-60.1(-69.4,-41.4)	-54.9(-74.2, -37.1)	-73.6(-86.2, -43.5)	-65.1(-78.8, -50.9)	0.070
	Thymic attenuation score, n(%)	n = 52	n = 21	n =18	n = 60	0.131
	Score 0	22(42.3%)	6(28.6%)	12(66.7%)	33(55%)	
	Score 1	26(50%)	13(61.9%)	4(22.2%)	22(36.7%)	
	Score 2	3(5.8%)	2(9.5%)	2(11.1%)	5(8.3%)	
	Score 3	1(1.9%)	0(0%)	0(0%)	0(0%)	
Male <45 years	Mean CT attenuation, HU, median (IQR)	-52.3(-61.6, -25.4)	-38.6(-52.6, -24.0)	-55.3(-73.7, -17.2)	-76.4(-85.1, -64.6)	<0.001
	Thymic attenuation score, n(%)	n = 37	n = 11	n = 10	n = 29	<0.001
	Score 0	6(16.2%)	2(18.2%)	3(30%)	24(82.8%)	
	Score 1	23(62.2%)	7(63.6%)	5(50%)	5(17.2%)	
	Score 2	8(21.6%)	2(18.2%)	2(20%)	0(0%)	
	Score 3	0(0%)	0(0%)	0(0%)	0(0%)	
Male ≥45 years	Mean CT attenuation, HU, median (IQR)	-53.8(-66.0, -44.2)	-52.7(-77.3, -17.7)	-63.7(-77.0, -41.9)	-75.0(-86.1, -66.3)	0.023
	Thymic attenuation score, n(%)	n = 10	n = 5	n = 6	n = 18	0.144
	Score 0	4(40%)	1(20%)	3(50%)	13(72.2%)	
	Score 1	6(60%)	4(80%)	3(50%)	5(27.8%)	
	Score 2	0(0%)	0(0%)	0(0%)	0(0%)	
	Score 3	0(0%)	0(0%)	0(0%)	0(0%)	

HU, Hounsfield units; SAPHO, synovitis, acne, pustulosis, hyperostosis, osteitis; RA, rheumatoid arthritis; AS, ankylosing spondylitis; HC, healthy controls.

**Figure 3:**
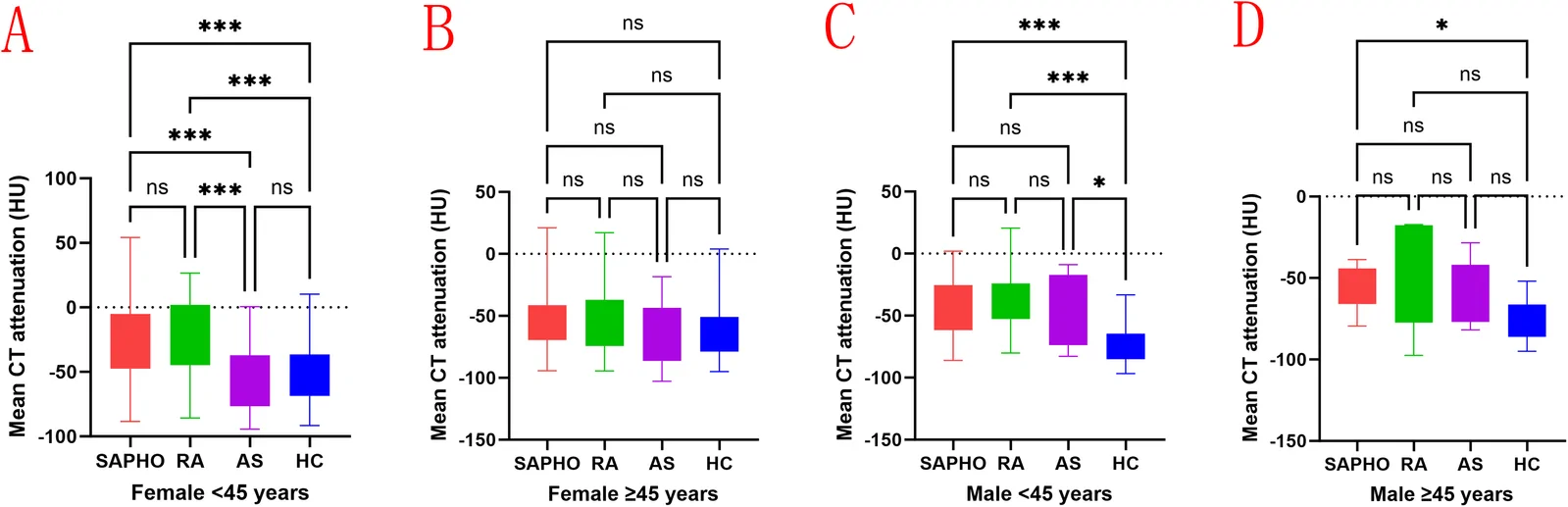
Sex- and age-stratified analysis of thymic CT attenuation values (HU) among the four groups. **(A)** Women <45 years: CT attenuation values were higher in the SAPHO and RA groups than in the AS and HC groups. **(B)** Women ≥45 years: no significant overall difference was observed among the four groups. **(C)** Men <45 years: CT attenuation values were higher in the SAPHO, RA, and AS groups than in HC. **(D)** Men ≥45 years: the overall difference was significant, and Bonferroni-adjusted pairwise analysis showed a significant difference only between SAPHO and HC. Age stratification used a cut-off of 45 years based on the mean age of complete fatty replacement of the thymus in the general population. Each panel shows box-and-whisker plots. ***Bonferroni-adjusted P<0.001, **P<0.01, *P<0.05; ns, not significant; HU, Hounsfield units; SAPHO, synovitis, acne, pustulosis, hyperostosis, osteitis; RA, rheumatoid arthritis; AS, ankylosing spondylitis; HC, healthy controls.

**Figure 4:**
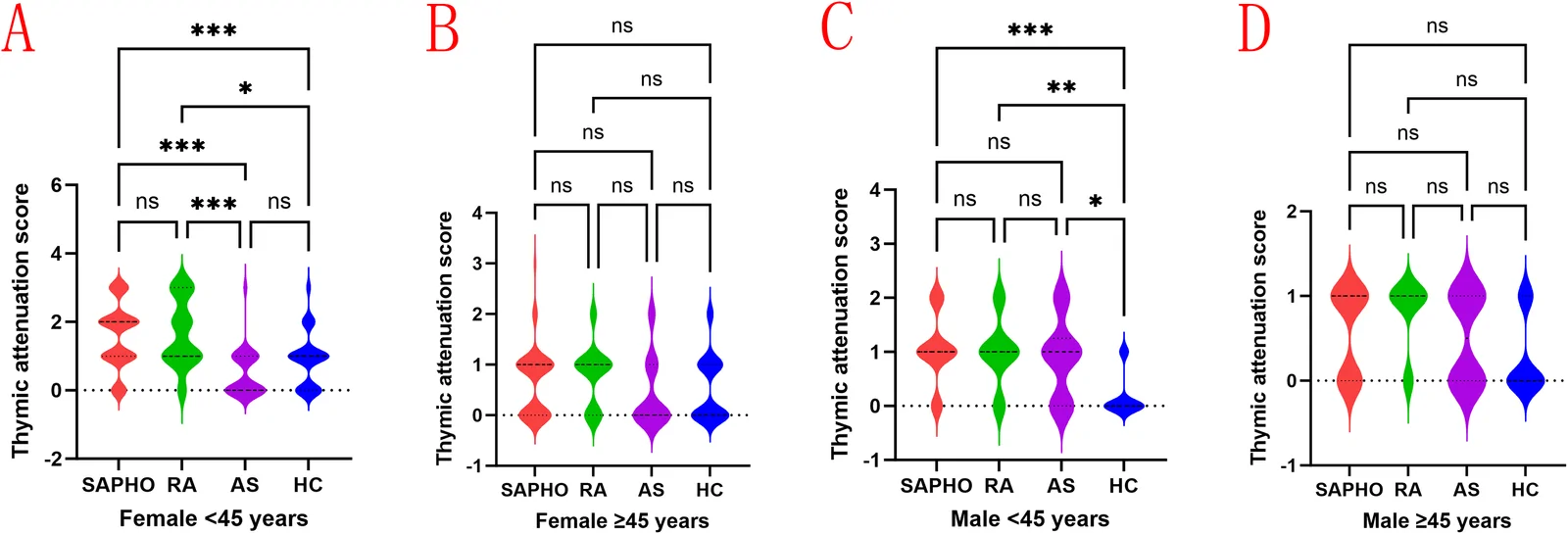
Sex- and age-stratified analysis of thymic attenuation scores (0–3) among the four groups. **(A)** Women <45 years: thymic attenuation scores were higher in the SAPHO and RA groups than in the AS and HC groups. **(B)** Women ≥45 years: no significant overall difference was observed among the four groups. **(C)** Men <45 years: thymic attenuation scores were higher in the SAPHO, RA, and AS groups than in HC. **(D)** Men ≥45 years: no significant overall difference was observed among the four groups. Age stratification used a cut-off of 45 years based on the mean age of complete fatty replacement of the thymus in the general population. Each panel shows violin plots. ***Bonferroni-adjusted P<0.001, **P<0.01, *P<0.05; ns, not significant; SAPHO, synovitis, acne, pustulosis, hyperostosis, osteitis; RA, rheumatoid arthritis; AS, ankylosing spondylitis; HC, healthy controls.

Among women aged <45 years, significant differences were observed across the four groups in both CT attenuation values (P<0.001) and attenuation score distribution (P<0.001). The proportion of women with higher attenuation (score ≥2) was notably high in the SAPHO group (54.3%, 44/81) and RA group (47.8%, 11/23), whereas 3.8% (1/26) of AS patients and 19.2% (14/73) of healthy controls in this age subgroup had higher attenuation (**Table 2**).

In contrast, among women aged ≥45 years, no significant differences were observed across the four groups in either CT attenuation values (P = 0.070) or attenuation score distribution (P = 0.131). The proportion of women with higher attenuation in this age subgroup was low across all groups.

Among men aged <45 years, significant differences were observed across groups in CT attenuation values (P<0.001) and attenuation score distribution (P<0.001). The proportion of men with higher attenuation was 21.6% (8/37) in the SAPHO group, 18.2% (2/11) in the RA group, 20.0% (2/10) in the AS group, and 0% (0/29) in the HC group.

Among men aged ≥45 years, CT attenuation values differed significantly across the four groups (P = 0.023), whereas the overall comparison of attenuation score distribution was not significant (P = 0.144). Bonferroni-adjusted pairwise comparisons showed a significant difference in CT attenuation values only between the SAPHO and HC groups. The proportion of men with higher attenuation was 0% across all groups in this age subgroup.

### Association between higher thymic attenuation and clinical characteristics in the SAPHO group

Within the SAPHO group, patients with higher thymic attenuation (score ≥2) were significantly more likely to be female (85.7% vs. 68.5%, P = 0.015) and younger (median age 37.5 years vs. 44 years, P<0.001) compared with those with lower attenuation (score 0–1). No significant differences were observed for disease duration, smoking status, alcohol consumption, elevated CRP, or elevated ESR between the two subgroups (P>0.05). Regarding symptom types, the distribution of cutaneous lesions, bone pain, or both did not differ significantly between the higher and lower attenuation groups (P = 0.697) (**Table 3**).

**Table 3:** Comparison of clinical characteristics between SAPHO patients with higher and lower thymic attenuation.

Variable	Higher attenuation (Score 2-3) (n=56)	Lower attenuation (Score 0-1) (n=124)	*P*
Female, n(%)	48(85.7%)	85(68.5%)	0.015
Age, years, median (IQR)	37.5(34, 41)	44(39, 53.8)	<0.001
Symptom type, n (%)			0.697
Cutaneous lesions	18(32.1%)	37(29.8%)	
Bone pain	1(1.8%)	5(4.0%)	
Both	37(66.1%)	82(66.1%)	
Disease duration, months, median (IQR)	24(6, 48)	24(12, 60)	0.434
Smoking, n (%)	23(41.1%)	54(43.5%)	0.756
Alcohol consumption, n (%)	9(16.1%)	18(14.5%)	0.787
ESR elevated, n (%)	9(16.1%)	34(27.4%)	0.098
CRP elevated, n (%)	7(12.5%)	19(15.3%)	0.618

ESR, erythrocyte sedimentation rate; CRP, C-reactive protein.

## Discussion

This study provides the first systematic evaluation of thymic CT imaging findings in patients with SAPHO syndrome, with comparisons to patients with RA, AS, and healthy controls. The main findings are as follows: (1) the prevalence of higher thymic attenuation (score ≥2) was 31.1% in SAPHO and 25.0% in RA, compared with 8.3% in AS and 10.6% in HC; (2) *post hoc* analyses of the ordinal attenuation score and CT attenuation values demonstrated a consistent pattern in which SAPHO and RA showed similarly higher thymic attenuation than AS and HC, while SAPHO did not differ significantly from RA and AS did not differ from HC; and (3) within the SAPHO group, higher attenuation was significantly associated with female sex and younger age.

### Thymic imaging findings in SAPHO syndrome

SAPHO syndrome is characterized by aseptic inflammatory osteitis, synovitis, and cutaneous lesions, with the Th17/IL-17 pathway implicated in its pathogenesis (10, 13). As the primary site of T-cell development and selection, the thymus is biologically relevant to immune tolerance; however, CT attenuation alone cannot establish thymic immune dysfunction. In the present study, the prevalence of higher thymic attenuation was numerically highest in the SAPHO group (31.1%) but did not differ significantly from the RA group on *post hoc* comparisons. These findings support the presence of a thymic imaging phenotype in SAPHO that is broadly similar to that observed in RA.

The prevalence of higher thymic attenuation in SAPHO syndrome (31.1%) was also numerically higher than proportions previously reported in RA (12.9%) (4), SSc (9.5%) (5), pSS (15.3%) (7), and PM/DM (19.1%) (7). However, direct comparison across studies should be interpreted cautiously because of differences in age distributions, disease characteristics, treatment exposure, and imaging methods. The relatively high prevalence observed in the present cohort nevertheless supports further investigation of thymic imaging findings in SAPHO syndrome.

Within the SAPHO group, higher thymic attenuation was not significantly associated with symptom type, disease duration, elevated CRP, or elevated ESR. Thus, the present data do not support interpretation of thymic attenuation as a marker of inflammatory disease activity. Its clinical significance requires further study using validated activity measures and longitudinal imaging.

### Comparison with rheumatoid arthritis

Murata et al. (4) reported that the proportion of patients with higher thymic attenuation (score ≥2) in RA was 12.9%, with a strong association with anti-citrullinated protein antibodies (ACPA) (odds ratio = 15.2). In the present study, the proportion of RA patients with higher attenuation was 25.0%, which is higher than that reported by Murata et al. This discrepancy may be partially explained by differences in patient age. In the study by Murata et al., the median age of RA patients was 67 years (IQR, 57–74 years), whereas in the present study, the median age of RA patients was 42 years (IQR, 36–50 years). Given that thymic attenuation is known to decrease with advancing age (8, 9), the younger age of the RA cohort in the present study may have contributed to the higher observed proportion of higher thymic attenuation. Additional factors, such as differences in ethnicity (Japanese vs. Chinese), disease duration, or treatment regimens, may also have played a role.

CT attenuation values were numerically higher in the RA group than in the SAPHO group (median −37.9 HU vs. −44.6 HU), but the difference was not statistically significant. Likewise, the ordinal attenuation scores did not differ between the two groups. Both groups showed a greater frequency of higher attenuation in younger participants and in women, suggesting a shared sex- and age-related imaging pattern rather than a disease-specific difference between SAPHO and RA.

### Comparison with ankylosing spondylitis

A notable finding of the present study was that thymic CT findings in AS were similar to those in healthy controls. The prevalence of higher attenuation was 8.3% in AS and 10.6% in HC, and *post hoc* analyses showed no significant difference between these groups in either the ordinal attenuation score or CT attenuation values. Although both AS and RA are chronic inflammatory rheumatic diseases, their immunopathogenesis differs substantially. AS is characterized predominantly by axial and entheseal inflammation and is strongly associated with IL-23/IL-17-related pathways, whereas RA is characterized by synovial inflammation and is associated with autoantibodies such as ACPA and rheumatoid factor (15, 16). The present findings indicate that the thymic CT phenotype observed in SAPHO and RA was not evident in AS, although imaging data alone cannot establish the presence or absence of thymic involvement in disease pathogenesis.

This contrast may reflect differences in the mechanisms underlying disease initiation and maintenance. RA and SAPHO syndrome may involve immune processes related to T-cell selection and tolerance, whereas AS has strong peripheral components such as gut microbiota, enthesitis, and mechanical stress (17, 18). The biological effects of the IL-17 pathway are also context dependent across tissues, as illustrated by reports of colitis induced by IL-17A inhibitors (11). These mechanistic interpretations remain hypotheses and require direct immunological and pathological validation.

Of note, subgroup analysis revealed that 20.0% of young male AS patients (<45 years) exhibited higher thymic attenuation, whereas none of the healthy controls in this subgroup did (**Table 2**). Given the small number of young male AS patients (n=10), this finding should be interpreted cautiously and does not alter the overall observation that AS and HC showed similar thymic CT findings at the group level.

### Sex and age characteristics: relation to normal thymic involution and MG

Ackman et al. (8) reported that women aged 20–30 years have significantly higher thymic attenuation than men of the same age and more frequently exhibit a quadrilateral morphology. In the present study, women accounted for 85.7% of SAPHO patients with higher thymic attenuation, and the median age of this subgroup was 37.5 years. More importantly, among women aged <45 years, higher attenuation was observed in 54.3% of SAPHO patients compared with 19.2% of age- and sex-comparable healthy controls. This excess relative to healthy controls is compatible with relative persistence of thymic soft tissue or delayed involution, although the cross-sectional CT findings cannot establish a pathological involution process.

The between-group differences appeared most pronounced among women aged <45 years. In this subgroup, higher thymic attenuation was observed in 54.3% of SAPHO and 47.8% of RA patients, compared with 3.8% of AS and 19.2% of HC participants (**Table 2**). Among women aged ≥45 years, higher attenuation was uncommon and no significant between-group differences were observed. Among men aged <45 years, higher attenuation occurred in approximately 18–22% of the three disease groups but was absent in HC, whereas no men aged ≥45 years had a score ≥2. These findings suggest that sex and age modify the observed thymic CT pattern; however, the subgroup analyses were exploratory and no formal interaction test was performed.

Naik et al. (9) reported a mean age of 45.4 years for complete fatty replacement of the thymus in the general population. The median age of 37.5 years among SAPHO patients with higher attenuation is therefore not, by itself, evidence of abnormal involution. Rather, the comparison with age- and sex-matched healthy controls supports the interpretation of relatively greater persistence of thymic soft tissue in a subset of SAPHO patients. Similar age-related persistence of thymic tissue has been described in SSc (5, 6), RA (4), and PM/DM (7).

Berrih-Aknin and Le Panse (3) reported that thymic follicular hyperplasia in MG predominantly affects women, with a peak onset between 20 and 40 years of age. The predominance of higher thymic attenuation among younger women in SAPHO and RA raises the possibility that sex-related or hormonal factors may influence thymic imaging findings. Estrogen has been shown to affect immune tolerance through mechanisms including downregulation of AIRE expression (19). However, the present study did not assess hormonal status or thymic immune function, and therefore cannot establish an estrogen-dependent mechanism.

Thymic follicular hyperplasia in MG is characterized pathologically by germinal center formation, B-cell infiltration, and expansion of lymphoid tissue. Such changes could theoretically contribute to increased soft-tissue attenuation on CT, but CT attenuation cannot determine the underlying histological substrate. Accordingly, whether higher thymic attenuation in SAPHO or RA corresponds to lymphoid follicular hyperplasia or germinal center formation remains speculative and requires imaging–pathology correlation.

## Limitations

Several limitations of this study should be acknowledged. First, the retrospective design precludes the exclusion of potential effects of treatments (e.g., glucocorticoids, csDMARDs, biologics, or targeted therapies) on thymic imaging findings, although treatment exposure at the time of CT was recorded and is summarized in **Table 1**. Second, the absence of pathological correlation means that the correspondence between imaging-defined “higher attenuation” and specific histological changes remains speculative. Third, the RA and AS groups were relatively small (60 patients each), and some sex- and age-stratified subgroups, particularly men aged ≥45 years, contained very few participants. These subgroup analyses should therefore be considered exploratory. Finally, the cross-sectional design does not establish whether higher attenuation represents delayed involution, stable residual thymic tissue, or a reversible change over time.

## Conclusions

Thymic CT attenuation scores and CT attenuation values were higher in patients with SAPHO syndrome and rheumatoid arthritis than in patients with ankylosing spondylitis and healthy controls, whereas SAPHO and RA showed similar thymic CT findings. The differences appeared most pronounced among women aged <45 years, and higher attenuation within SAPHO was associated with female sex and younger age. Increased thymic CT attenuation may represent an imaging phenotype associated with SAPHO syndrome and RA, but the present data do not support its use as a marker of disease activity. Further longitudinal studies incorporating immunological and pathological correlation are needed to clarify its biological and clinical significance.

## Data Availability

The original contributions presented in the study are included in the article; further inquiries can be directed to the corresponding author.
